# Worry and Anxiety in the Era of Early Diagnosis and Disease Modifying Treatment for Alzheimer’s Disease

**DOI:** 10.1007/s11920-026-01704-1

**Published:** 2026-07-27

**Authors:** Shana D. Stites, Katherine Lee, Carolyn Kuz, David Mandell, Hillary R. Bogner, Rebecca Brown

**Affiliations:** 1 Department of Psychiatry, Perelman School of Medicine, University of Pennsylvania, Philadelphia, PA 19104 USA; 2 Division of Geriatric Medicine, Department of Medicine, Perelman School of Medicine, University of Pennsylvania, Philadelphia, PA 19104 USA; 3 Department of Family Medicine and Community Health, Perelman School of Medicine, University of Pennsylvania, Philadelphia, PA 19104 USA

**Keywords:** Anxiety, Alzheimer’s disease, Biomarker testing, Dementia worry, Diagnosis, Testing

## Abstract

**Purpose of Review:**

Psychologists and psychiatrists are well-positioned to be at the forefront of addressing the challenges posed by anxiety in early diagnosis and treatment of Alzheimer’s disease (AD) dementia that are unfolding in primary care settings. The present article provides a summary of emerging areas in Alzheimer’s disease testing and research to guide clinical decisions and research pursuits.

**Recent Findings:**

The present article provides an overview of how anxiety is understood in early diagnosis of dementia due to AD, its influence in AD-related mechanisms, and the implications of these findings on current research practices.

**Summary:**

We conducted focused literature reviews between January and June 2026 and synthesized the information to provide a narrative review. Overall, the clinical roles of anxiety in AD dementia are expanding— from being viewed primarily as reactions to cognitive decline to serving as informative features of the diagnostic process and clinical course of the disease.

## Introduction

Advances in biomarkers of Alzheimer’s disease (AD) are making early diagnosis and disease-slowing treatments possible for dementia [1–13]. As early diagnosis and treatment of AD dementia become more readily available in routine care, people who develop disease due to AD pathology will have the chance to live longer and better, with less cognitive and functional impairment impacting their daily lives [14–18]. Notably, the success of these efforts hangs in large part on understanding and addressing anxiety.

Early diagnosis is paramount to the success of disease-slowing therapies for AD pathology [4, 19–21]. Yet anxiety can complicate diagnosis as people may seek diagnosis because of anticipatory worry when they do not have dementia symptoms. If not properly addressed, these worries can lead to diagnosis shopping [22, 23], avoidable healthcare costs [24–30], and lower well-being [31]. To further complicate the matter, emerging evidence suggests that anxiety may be part of the underlying mechanisms associated with AD dementia [32–35], indicating a need to more deeply understand the types of anxiety experienced by individuals seeking early AD diagnosis. The potential for clinical interpretation of anxiety to impact use of the new therapies warrants careful attention, as new therapies have the potential to improve individuals’ well-being [19, 36, 37], but can also pose serious health risks, such as amyloid-related imaging abnormalities (ARIA) [38, 39].

Psychologists and psychiatrists are well-positioned to be at the forefront of addressing the challenges posed by anxiety in early diagnosis and treatment of AD dementia, given their clinical expertise in caring for patients with health worries and advancing research to understand mechanisms involving anxiety. Moreover, they deliver care across almost all clinical settings [40, 41]. That knowledge and influence is essential to support the translation of the new advances in AD, which requires the adaptation and coordination of services across primary care, specialty care, and community settings.

The present article provides an overview of how anxiety is currently understood in early diagnosis of dementia due to AD, its influence in AD-related mechanisms, and implications for current research practices. This focus complements that of prior commentaries and guidelines for use of AD blood tests in patients with cognitive impairment [42–49] and for addressing AD genetic test results [50]. We conducted focused literature reviews between January and June 2026 and synthesized the information to provide a narrative review. Our goal is to provide a summary for clinicians to bring attention to this emerging area as well as offer the latest evidence to guide clinical decisions and research pursuits, while recognizing these areas will continue to evolve.

## Anxiety’s Influence in the Diagnostic Process

Many people worry about developing dementia [29, 51–55]. Often, these worries are about Alzheimer’s rather than other lesser known causes of dementia [56–59]. In some cases that worry is sufficient to motivate them to seek evaluation, in contrast to individuals who are encouraged by their family members to seek care or who seek care because they have become less able to carry out major tasks in daily life. The reasons prompting the worry often include interpretation of age-related changes in cognition as early signs of dementia, a family history placing them at increased risk of developing dementia, or the influence of advertising from direct-to-consumer testing companies.

We expect that people who are worried about developing dementia will increasingly seek out evaluation for AD because they hear about “new tests” for AD. In primary care, about 1 in every 10 patients has dementia [60], and yet most (70%) physicians report being asked about Alzheimer’s or dementia at least once a week [60]. Moreover, the recently approved blood tests for AD biomarkers are garnering significant public attention through advertising for direct-to-consumer testing. There is a need to more deeply understand the types of anxiety experienced by individuals seeking early AD diagnosis. Unaddressed anticipatory worry and misattributed disease-related anxiety have the potential to lead to inferior outcomes.

### Why Alzheimer’s Biomarker Testing is not a Solution to Dementia-related Worry

**Fig. 1:**
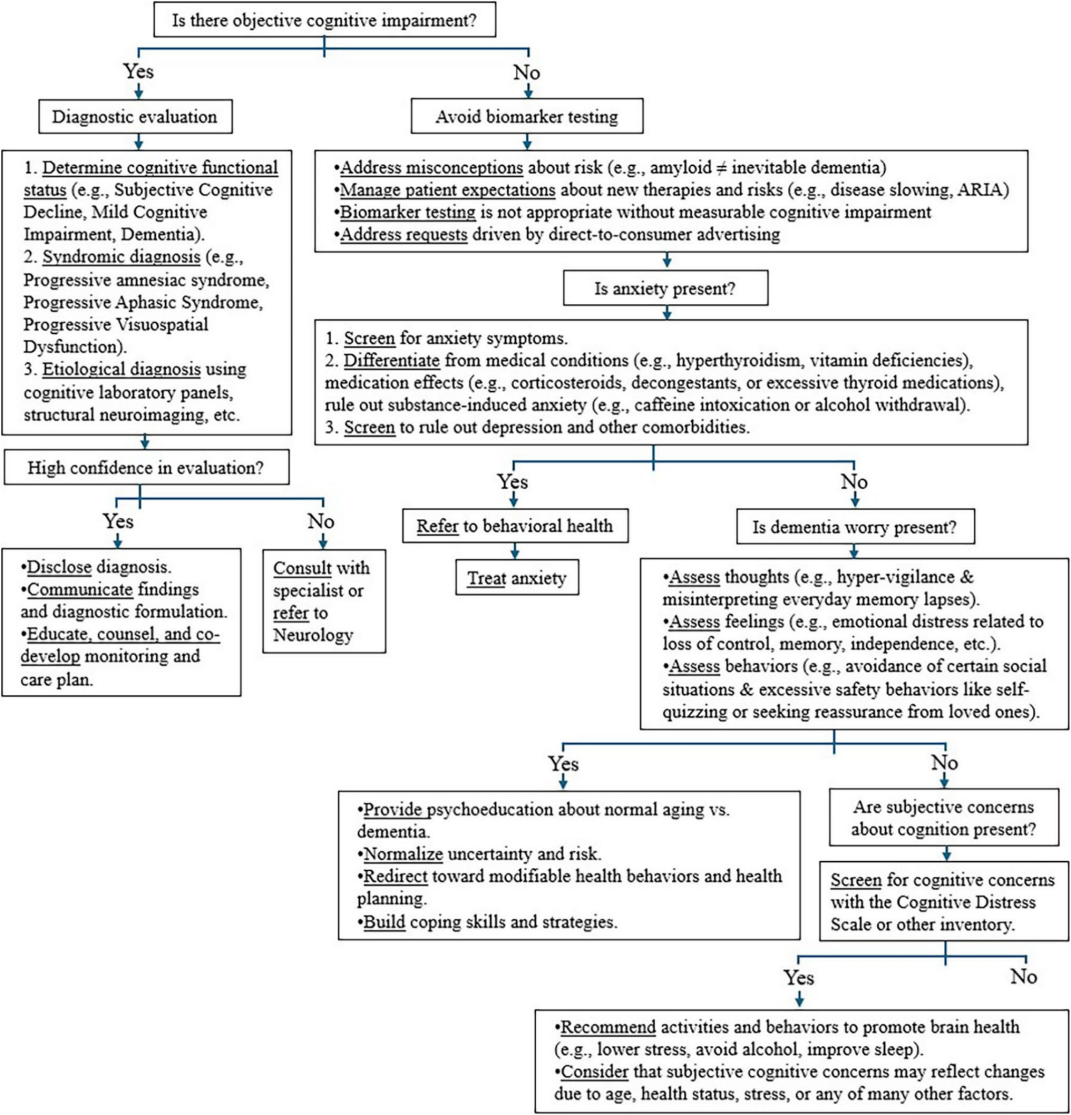
Flow for clinical decision making in persons with and without cognitive impairment

Individuals may seek evaluation for cognitive concerns or AD dementia in order to access testing for AD biomarkers. The recent approval of blood tests for Alzheimer’s biomarkers by the U.S. Food and Drug Administration (FDA) has led to a major expansion in the availability of this testing, moving it from specialty offices to primary care settings. Despite wide availability, appropriate use recommendations limit AD biomarker testing to patients with cognitive impairment [61–63], which refers to persistent, measurable deficits in memory and thinking that exceed normal age-related changes [64]. The flow for clinical decision making in persons with and without cognitive impairment is shown in Fig. 1.

People without cognitive impairment may have interest in this testing because they are worried about developing dementia [65]. Yet, AD biomarker tests do not indicate if a patient has symptoms or will develop symptoms. Longitudinal studies indicate that a substantial percentage—ranging from roughly 30% to 50%—of cognitively unimpaired individuals with elevated brain amyloid did not develop dementia symptoms after 10 years [66–69].

In addition, research is ongoing to understand the performance of AD biomarkers in populations outside those that comprised the samples used to develop the testing, which were fairly homogeneous. Consequential differences in the performance of these tests have been observed between social groups, including those defined by race [70]. Certain comorbidities can also affect the performance of AD biomarker tests [71].

While people experiencing persistent or more serious cognitive concerns, such as getting lost in familiar places [72], may benefit from further evaluation for cognitive impairment (for reviews see [48, 49]), the care pathway for those worried about dementia who do not have cognitive impairment is less clear, and evolving as new therapies for use in earlier disease are tested [12, 19, 73]. Many individuals without cognitive impairment may have concerns about their memory and thinking, as changes in cognition can occur with aging [74], present as a general symptom of many conditions [75], and create difficulties that lower quality of life [76]. Currently, these concerns are not sufficient to indicate use of AD tests and cannot be addressed by the results of that testing.

### Why Unresolved Worry after Exclusion from Alzheimer’s Testing is a Problem

Anxiety, defined broadly as worries related to everyday stressors, usually on its own does not necessitate clinical intervention [77]. These worries typically lead to short-term physiological and emotional reactions that dissipate when the situation resolves. However, when concerns lead a person to seek formal evaluation, the worries may reflect a type of health anxiety, defined as persistent, overwhelming fear of having or developing a serious medical condition, such as dementia.

Dementia worry is the emotional response to the perceived threat of developing dementia. These worries in older adults about developing dementia are often connected to their knowledge of having a family history for dementia, a genetic disposition that conveys an elevated risk, or both [50, 78]. While clinicians may be able to reassure individuals that Alzheimer’s biomarker testing would not answer their question as to whether they might develop dementia, brief interventions for anxiety may also be needed to effectively reduce dementia-related worry.

Psychoeducational interventions that provide facts about AD-related tests and dementia may help address anticipatory worry about dementia and improve clinical outcomes. For instance, studies examining psychological and socio-psychological outcomes in AD prevention trials have shown that disclosure programs that accompany return of AD biomarker results to participants may reduce worry, stigma and other poor socio-psychological outcomes [79–82]. Interestingly, the improvement in outcomes has been observed in groups learning either a positive or negative result, suggesting it is more about the uncertainty than a specific type of test result.

For individuals with cognitive concerns, alleviating anxiety may help reduce the distress associated with those concerns, even in people without cognitive impairment [76]. In addition, reducing anxiety is essential for achieving benefits of memory training interventions [83]. Patients with lower confidence in their abilities are more prone to these emotional states, which directly diminishes their overall well-being [84].

## Roles of Anxiety in Clinical Pathways/Mechanisms

Anxiety’s role in dementia has historically received relatively little attention and significantly less attention than depression [33, 85]. Along with depression, anxiety has long been grouped with "behavioral and psychological symptoms of dementia" (BPSD), which affect up to 90% of patients with dementia [86–89]. Historically, anxiety and other mood symptoms have been understood to reflect a multifactorial etiology involving biological, psychological, and social factors, and addressed by clinicians as reactions to cognitive decline [32, 33, 90–93].

More recent research suggests that anxiety along with depression may be factors in the causal model of AD dementia. A new hypothesis is emerging that anxiety and depression are not competing causal explanations for cognitive concerns, but rather clinical manifestations of AD pathology that may contribute to the cognitive concerns themselves [94, 95]. In other words, the clinical roles of anxiety and depression are expanding from a focus on management of these symptoms as reactions to cognitive decline to understanding their roles as informative in the diagnostic process.

The conceptualization of anxiety and depression as manifestations along a shared continuum of distress is established in the psychological literature [96–98]. The emerging research in AD dementia builds on this understanding to suggest that anxiety, in subclinical forms, may serve as a marker of neurodegeneration, which could be determined via AD biomarker testing to be occurring in the context of AD pathology [83, 94, 99, 100]. This hypothesis is important to investigate further because if subclinical anxiety is a signal of disease progression, it could aid in distinguishing people with progressive disease from the many individuals with AD pathology who never develop dementia.

### Anxiety Symptoms in Clinical Presentations of AD Dementia

Anxiety symptoms are prevalent during the late preclinical phase of AD, which occurs before objective cognitive impairment is measurable [83]. This stands in contrast to other common symptom patterns reported to date in dementia-focused research, such as those that show mood symptoms emerging after onset of cognitive impairment (Table 1) [101, 102]. In the late preclinical phase, anxiety may peak when the individual is aware of their declining abilities and emerging problems with their cognition are beginning to outstrip their coping mechanisms [83, 84].

**Table 1:** Anxiety and depression surrounding preclinical (asymptomatic) concerns and dementia

Category description	Description
Depression as a precursor to cognitive decline	A history of major depressive episodes before the age of 60 is strongly associated with the later development of MCI and more severe, treatment-resistant emotional distress in later life [102]. In this context, depression serves as a modifiable risk factor that may precede and accelerate the rate of cognitive decline [124]
Early-stage anxiety in cognitive decline	Anxiety symptoms are frequently prevalent during the subjective memory complaints stage, which is considered a late preclinical phase of Alzheimer’s disease occurring before objective cognitive impairment is measurable [83]. Anxiety increases the risk of progression from MCI to dementia [35, 103, 104], potentially associated with the neurodegenerative process [94]
Depression as an early manifestation of AD	Depression is identified as a common early manifestation of the Alzheimer’s process, appearing early in the decline and persisting as the dementia becomes more severe [125–129]
Decline-induced emotional distress	Once MCI or mild dementia is established, individuals face a significantly increased risk of developing new onset depression and anxiety—with rates reaching up to 43% compared to only 20% in those with normal cognition [130]. This suggests that the loss of autonomy and cognitive function can trigger or exacerbate these emotional states [91, 129]

*MCI* Mild Cognitive Impairment; *AD* Alzheimer’s disease

What is not yet understood is how anxiety may operate in people who are experiencing changes due to AD pathology and are still compensating for the emerging problems in their cognition. It remains unclear whether this compensatory phase—during which individuals maintain functional performance despite emerging cognitive changes—overlaps with help-seeking driven by anticipatory anxiety, or whether its defining features could be leveraged to identify candidates for disease-modifying therapies earlier in the disease course. It is plausible that anxiety could increase the risk of progression from no clinical symptoms to early impairment as anxiety is known to increase the risk of progression between other stages of dementia [35, 103, 104].

The emerging research in dementia that suggests anxiety may correspond to neurodegeneration is synergistic with findings from psychological studies. For example, individuals with anxiety that show a reduced responsiveness to psychotherapy may have underlying neuroinflammation [102], which has been linked to neurodegeneration [105]. In another line of AD-related research, anxiety that was associated with food insecurity, which is strongly linked to neuroinflammation [106–108], was associated with cognitive concerns [109]. Together, these data support the importance of better understanding how anxiety may act as part of pathways in neurodegeneration. In the longer term, understanding how anxiety corresponds with neurodegeneration may help support efforts to move AD diagnosis earlier in the disease process [17, 110, 111].

## Implications for Measuring Anxiety and Depression in Early AD Research

To understand whether anxiety and depression are currently being measured sufficiently to lead to advances in understanding of their effects in early AD, we aimed to determine whether studies tend to capture the full character and continuum of the severity of these mood symptoms. We examined screening and research protocols for trials of early AD (i.e., those that included or excluded participants based on amyloid status) that were funded by the National Institute on Aging (NIA). Of 193 active, NIA funded trials, nine used amyloid status to determine the inclusion or exclusion of research participants.

In all of the nine trials, participants who had elevated anxious or depressive symptoms and those with elevated risk of suicide were excluded from trial participation based on a lack of psychological readiness. Psychological readiness in Alzheimer's research is the emotional and mental stability of a participant or patient to process complex, potentially distressing medical information without suffering undue anxiety, depression, or distress [112]. Psychological readiness is emphasized in AD trials, particularly in those that involve returning the results of AD gene or biomarker testing [113–115].

While anxiety and depression measures are often included as secondary or exploratory outcome measures to track progression of neuropsychiatric symptoms as part of the potential response to treatment, psychological readiness practices in these trials may limit the discoveries associated with these measures. The exclusion of individuals with certain symptoms or levels of anxiety and depression from the trials for purposes of safety may restrict the range of scores and limit the generalizability of findings. It is unclear whether these safety practices are prudent or overly cautious. Emerging data on socio-psychological outcomes from these trials suggest that programs disclosing AD results may, contrary to concerns about increasing distress, actually lower socio-psychological concerns [81, 82]. Focused studies of individuals deemed too high risk for these trials may help understand the implications of anxiety and depression in early AD diagnostic procedures and disease mechanisms.

In addition, anxiety and depression were measured in the nine trials using a small inventory of clinical tools that placed limitations on what can be understood about mood in early AD (Table 2). In brief, anxiety was measured using the State-Trait Anxiety Inventory (STAI) [116], the Beck Anxiety Inventory (BAI) [117], and The Hospital Anxiety and Depression Scale (HADS) [118] with the latter used for measuring both anxiety and depression. Key instruments for assessing depression were the Geriatric Depression Scale (GDS) [119], the Patient Health Questionnaire-9 (PHQ-9) [120], and the Beck Depression Inventory (BDI) [121]. For suicide risk, the Columbia-Suicide Severity Rating Scale (C-SSRS) [122] provided a structured protocol of plain-language questions to identify ideation and behavior. Finally, the Neuropsychiatric Inventory (NPI) [123], an informant-based interview, was used specifically to capture behavioral changes in patients with neurodegenerative disorders.

**Table 2:** Review of assessments for anxiety and depression in select clinical trials

Factor	GDS-15 [131–133]	NPS [123, 134]	STAI [135]	HADS [136–139]	PHQ-9 [120, 140, 141]
Purpose	Screening for depression, specifically for older adults	Measures psychological and behavioral symptoms of depression in persons with dementia and other conditions	Measures two types of anxiety: state anxiety, which is temporary and based on current feelings, and anxiety, which is long-term and tendency-based	Measures depression and anxiety	Measures depressive symptoms
Format	15-item self report survey (5–7 min)	Interview or questionnaire format (NPS-Q)	40-item self report survey (20 items for state; 20 items for trait)	14-item self report survey (7 items for depression; 7 items for anxiety	9-item self report survey
Qualitative description of content	15 “yes/no” questions about specific symptoms	12 behavioral and psychological symptoms rated on 3-point scale of Mild, Moderate, and Severe	Items rated on 4-point Likert scales from Not at all, Somewhat, Moderately, and Very much for state anxiety, and Almost never, Sometimes, Often, and Almost always for Trait anxiety	Items rated on 4-point Likert scale from 0 to 3	Items rated on 4-point scale of not at all, sometimes, often and nearly every day
Development sample	> 55 years old	Adults with a mean age of 81	Any age	> 16 years old	> 12 years old
Score range	0–15	Varies	20–80	0–21	0–27
Threshold(s) of clinical interpretation	Depression is ≥ 5. High sensitivity (0.89) and high specificity (0.77)	Neuropsychiatric symptoms is ≥ 1. High sensitivity (0.74) and specificity (0.80)	Anxiety is > 40. High sensitivity (0.80–0.90) and moderate specificity (0.57–0.77)	HADS-D or HADS-A “possible case” is ≥ 8; “probable case” is > 11 of either depression or anxiety. HADS has a high specificity (0.88) and sensitivity (0.87)	Depression is > 5 for mild, > 10 for moderate, > 15 for moderately severe, and > 20 for severe. High specificity (0.88) and high sensitivity (0.88)
Window of recall	Past 7 days	Past 4 weeks	State: current Trait: lifetime	Past 7 days	Past 2 weeks
Population applicability	Geriatric, dementia, and Parkinson’s disease	Dementia, Parkinson’s disease	Any; non-specified	Patients with physical illnesses. Somatic symptoms of depression are excluded from the total HADS score	Patients in primary care settings

*GDS* Geriatric Depression Scale; *NPI* Neuropsychiatric Inventory; *STAI* State-Trait Anxiety Inventory; *HADS* Hospital Anxiety and Depression Scale; *PHQ-9* Patient Health Questionnaire; *C-SSRS* Columbia-Suicide Severity Rating Scale

The measures differ in their thresholds for clinically relevant effects, observation windows and clinical interpretations, particularly for anxiety. These characteristics impact the effectiveness of the measures for identifying subclinical (i.e., low) levels of anxiety and depression. In fact, these instruments were developed for detecting major mood disorders, not subclinical levels. For example, the HADS has been shown to under-detect mild cases of anxiety and depression because its three-tier system lacks a direct category for subclinical depression, whereas the PHQ-9’s 4-point scale explicitly defines a mild range for depression. Moreover, the STAI demonstrates strong correlations with depressive symptom measures, limiting its discriminant validity in identifying subclinical anxiety. Consequently, while these tools are valuable, their precision in a subclinical context requires undertaking steps to adjust standard thresholds and account for the restricted range and statistical power of specific instruments.

Moreover, differences across the measures in how they do or do not assess somatic symptoms are likely to have a substantive impact on the sensitivity, specificity and character of the anxiety and depression that is identified. Measures like the HADS and GDS-15 exclude somatic symptoms like fatigue or weight change, making them more effective than the BAI for identifying subclinical affective symptoms in older adults without the interference of physical ailments. This suggests research may be needed to refine or develop assessments that can most effectively measure anxiety and depression for their relevance in early AD.

## Conclusion

This article provides an overview of how anxiety can present and is currently understood in early diagnosis of dementia due to AD, its influence in AD-related mechanisms, and implications for current research practices. Our goal is to provide a summary for psychologists, psychiatrists and other clinicians to bring attention to this emerging area as well as offer the latest evidence to guide informed decisions and research pursuits. Overall, the clinical roles of anxiety and depression in AD dementia are shifting—from being viewed primarily as reactions to cognitive decline to serving as informative features of the diagnostic process and clinical course of the disease.

Anxiety, in particular, is increasingly recognized as both a contributor to the socio-psychological context of early diagnosis and an interpretable symptom of AD mechanisms with specific clinical relevance. More research is needed to understand how to best support individuals who may be worried about developing AD dementia. Some may experience suboptimal socio-psychological outcomes, some may show increased anxiety as the first sign of progressive neurodegeneration, and still others may experience both. Individuals seeking early diagnosis and treatment for AD may be affected by varied types and levels of anxiety.

An essential part of advancing research on anxiety in early AD will require improving instruments to detect subclinical anxiety and depression. The substantial phenomenological and psychometric overlap between anxiety, depression, cognitive concerns and somatic complaints complicates the identification of subclinical symptom levels. Research is needed to determine how to balance diagnostic specificity and sensitivity to achieve these goals, as existing instruments are often not optimized to detect lower-severity symptom presentations.

It is essential that the investigations into the types of anxiety in early AD reflect the endeavors of multidisciplinary research teams of psychologists, psychiatrists and primary care physicians. Psychologists and psychiatrists have a long history of theoretical inquiry and clinical care for anxiety. It would serve the interests for this history to inform efforts to understand the roles of anxiety in early AD and to collaborate closely with primary care physicians.

Given the level of worry about dementia in the public combined with the recent approval of blood testing for AD biomarkers for use in primary care and investment in advertising from direct-to-consumer companies, many people may seek out this testing. Yet, this testing may benefit only a small subgroup. It is essential clinicians can address worry about dementia in the absence of testing and help support individuals who may be able to improve their cognition by lowering their anxiety. Moreover, discerning worry about dementia from subclinical anxiety that could indicate neurodegeneration would be a major advance in early identification of disease progression, which is essential to the success of new treatments for Alzheimer’s disease that work by slowing progression. To achieve these goals, research practices, including measurement of anxiety, may need to change.

## Key References

- Alzheimer’s Association [53]:
  - ○ The results of this survey found that 68% of Americans age 40 and older worried about developing Alzheimer’s disease or related dementias.
- Sun et al. [35]:
  - ○ This article provides evidence that anxiety increases the risk for cognitive progression in individuals without dementia. It also identifies a link between anxiety and pathology of axon-synapse injury and energy metabolism imbalance, suggesting anxiety may be part of the underlying mechanisms associated with AD dementia.
- Stites et al. [81]:
  - ○ This article demonstrates that disclosing Alzheimer’s disease biomarker results to participants may reduce stigma.
- Yin et al. [83]:
  - ○ This article reports that lower anxiety is associated with changes in the amygdala-hippocampal pathway, suggesting that anxiety, in subclinical forms, may serve as a marker of neurodegeneration.
- Stites et al. [94]:
  - ○ This article demonstrates that anxiety may be associated, generally, with neurodegeneration while depression may be associated with Alzheimer’s pathology.

## Data Availability

No datasets were generated or analysed during the current study.

## References

[1] Boxer AL, Sperling R. Accelerating Alzheimer’s therapeutic development: the past and future of clinical trials. Cell. 2023;186:4757–72. 10.1016/j.cell.2023.09.023. 37848035 10.1016/j.cell.2023.09.023 PMC10625460

[2] Cummings J, Apostolova L, Rabinovici GD, et al. Lecanemab: Appropriate use recommendations. J Prev Alzheimers Dis. 2023;10:362–77. 10.14283/jpad.2023.30. 37357276 10.14283/jpad.2023.30 PMC10313141

[3] Fu W, Ho PC-L. Blood-based biomarkers for Alzheimer’s disease: advances in early detection and monitoring of age-related neurodegeneration. Ageing Res Rev. 2026;117:103058. 10.1016/j.arr.2026.103058. 41667028 10.1016/j.arr.2026.103058

[4] Jack CR, Bennett DA, Blennow K, et al. NIA-AA research framework: toward a biological definition of Alzheimer’s disease. Alzheimers Dement J Alzheimers Assoc. 2018;14:535–62. 10.1016/j.jalz.2018.02.018. 10.1016/j.jalz.2018.02.018 PMC5958625 29653606

[5] Kim KY, Shin KY, Chang K-A. GFAP as a potential biomarker for Alzheimer’s disease: a systematic review and meta-analysis. Cells. 2023;12:1309. 10.3390/cells12091309. 37174709 10.3390/cells12091309 PMC10177296

[6] Olsson B, Lautner R, Andreasson U, et al. CSF and blood biomarkers for the diagnosis of Alzheimer’s disease: a systematic review and meta-analysis. Lancet Neurol. 2016;15:673–84. 10.1016/S1474-4422(16)00070-3. 27068280 10.1016/S1474-4422(16)00070-3

[7] Ossenkoppele R, van der Kant R, Hansson O. Tau biomarkers in Alzheimer’s disease: towards implementation in clinical practice and trials. Lancet Neurol. 2022;21:726–34. 10.1016/S1474-4422(22)00168-5. 35643092 10.1016/S1474-4422(22)00168-5

[8] Parums DV. Editorial: Real-world outcomes of disease-modifying therapies highlight the need for diagnostic biomarkers in early Alzheimer’s disease. Med Sci Monit Int Med J Exp Clin Res. 2025;31:e951655. 10.12659/MSM.951655. 10.12659/MSM.951655 PMC12499630 41030020

[9] Rani S, Dhar SB, Khajuria A, et al. Advanced overview of biomarkers and techniques for early diagnosis of Alzheimer’s disease. Cell Mol Neurobiol. 2023;43:2491–523. 10.1007/s10571-023-01330-y. 36847930 10.1007/s10571-023-01330-y PMC11410160

[10] van Oostveen WM, de Lange ECM. Imaging techniques in Alzheimer’s disease: A review of applications in early diagnosis and longitudinal monitoring. Int J Mol Sci. 2021;22:2110. 10.3390/ijms22042110. 33672696 10.3390/ijms22042110 PMC7924338

[11] VandeVrede L, Schindler SE. Clinical use of biomarkers in the era of Alzheimer’s disease treatments. Alzheimers Dement. 2024;21:e14201. 10.1002/alz.14201. 39740074 10.1002/alz.14201 PMC11775455

[12] Wu J, Geng C, Liu L, et al. Advancement of disease-modifying therapy of Alzheimer’s disease: from the perspective of new revised criteria for diagnosis and staging of Alzheimer’s disease. Med Plus. 2025;2:100112. 10.1016/j.medp.2025.100112.

[13] Xu C, Zhao L, Dong C. A review of application of Aβ42/40 ratio in diagnosis and prognosis of Alzheimer’s disease. J Alzheimers Dis. 2022;90:495–512. 10.3233/JAD-220673. 36155521 10.3233/JAD-220673

[14] Dubois B, Padovani A, Scheltens P, et al. Timely diagnosis for Alzheimer’s Disease: A literature review on benefits and challenges. J Alzheimers Dis. 2015;49:617–31. 10.3233/JAD-150692. 10.3233/JAD-150692 PMC4927869 26484931

[15] Gauthier SG. Alzheimer’s disease: the benefits of early treatment. Eur J Neurol. 2005;12(Suppl 3):11–6. 10.1111/j.1468-1331.2005.01322.x. 16144532 10.1111/j.1468-1331.2005.01322.x

[16] Porsteinsson AP, Isaacson RS, Knox S, et al. Diagnosis of early Alzheimer’s disease: clinical practice in 2021. J Prev Alzheimers Dis. 2021;8:371–86. 10.14283/jpad.2021.23. 34101796 10.14283/jpad.2021.23 PMC12280795

[17] Rasmussen J, Langerman H. Alzheimer’s disease – Why we need early diagnosis. Degener Neurol Neuromuscul Dis. 2019;9:123–30. 10.2147/DNND.S228939. 31920420 10.2147/DNND.S228939 PMC6935598

[18] Wessels AM, Dennehy EB, Dowsett SA, et al. Meaningful clinical changes in Alzheimer disease measured with the iADRS and illustrated using the Donanemab TRAILBLAZER-ALZ study findings. Neurol Clin Pract. 2023;13:e200127. 10.1212/CPJ.0000000000200127. 36891463 10.1212/CPJ.0000000000200127 PMC9987204

[19] Goswami S, Masurkar PP, Sherer JT. Donanemab and lecanemab in Alzheimer’s disease treatment: a narrative review of clinical trials and discussion of implications for patient access. Equity Neurosci. 2026;2:100057. 10.1016/j.neuros.2026.100057.

[20] Nakashima S, Sato K, Niimi Y, et al. Therapeutic time window of disease-modifying therapy for early Alzheimer’s disease. Alzheimers Dement Transl Res Clin Interv. 2025;11:e70102. 10.1002/trc2.70102. 10.1002/trc2.70102 PMC12122261 40453977

[21] Wu C-K, Fuh J-L. A 2025 update on treatment strategies for the Alzheimer’s disease spectrum. J Chin Med Assoc. 2025;88:495–502. 10.1097/JCMA.0000000000001252. 40442885 10.1097/JCMA.0000000000001252 PMC12637128

[22] Mosalisa M, Roomaney R. Medical gas-lighting, diagnostic odyssey and self-advocacy among women with premenstrual dysphoric disorder from nine countries. J Health Psychol. 2026. 10.1177/13591053251401286. 10.1177/13591053251401286 PMC13287426 41540801

[23] Sansone RA, Sansone LA. Doctor shopping. Innov Clin Neurosci. 2012;9:42–6. PMC3552465 23346518

[24] Institute of Medicine (IOM). Imperative: achieving greater value in health care. Best care low. Cost path contin. Learn. Health care am. Washington, DC: The National Academies Press; 2013.

[25] Müskens JLJM, Kool RB, van Dulmen SA, et al. Overuse of diagnostic testing in healthcare: a systematic review. BMJ Qual Saf. 2022;31:54–63. 10.1136/bmjqs-2020-012576. 10.1136/bmjqs-2020-012576 PMC8685650 33972387

[26] Norbye AD, Abelsen B, Førde OH, et al. Health anxiety is an important driver of healthcare use. BMC Health Serv Res. 2022;22:138. 10.1186/s12913-022-07529-x. 35109834 10.1186/s12913-022-07529-x PMC8812228

[27] Hanson L, Barclay T, Hanson A, et al. D2–4: failure on cognitive screening predicts increased healthcare utilization. Clin Med Res. 2014;12:86. 10.3121/cmr.2014.1250.d2-4.

[28] Boone KB. Fixed belief in cognitive dysfunction despite normal neuropsychological scores: neurocognitive hypochondriasis? Clin Neuropsychol. 2009;23:1016–36. 10.1080/13854040802441135. 18923966 10.1080/13854040802441135

[29] Kessler E-M, Bowen CE, Baer M, et al. Dementia worry: a psychological examination of an unexplored phenomenon. Eur J Ageing. 2012;9:275–84. 10.1007/s10433-012-0242-8. 28804427 10.1007/s10433-012-0242-8 PMC5549110

[30] Roberts JS. Anticipating response to predictive genetic testing for Alzheimer’s disease: a survey of first-degree relatives. Gerontologist. 2000;40:43–52. 10.1093/geront/40.1.43. 10750312 10.1093/geront/40.1.43

[31] Werner P, AboJabel H, Maxfield M. Conceptualization, measurement and correlates of dementia worry: a scoping review. Arch Gerontol Geriatr. 2021;92:104246. 10.1016/j.archger.2020.104246. 32980573 10.1016/j.archger.2020.104246

[32] Botto R, Callai N, Cermelli A, et al. Anxiety and depression in Alzheimer’s disease: a systematic review of pathogenetic mechanisms and relation to cognitive decline. Neurol Sci. 2022;43:4107–24. 10.1007/s10072-022-06068-x. 35461471 10.1007/s10072-022-06068-x PMC9213384

[33] Mendez MF. The relationship between anxiety and Alzheimer’s disease. J Alzheimers Dis Rep. 2021;5:171–7. 10.3233/ADR-210294. 33981954 10.3233/ADR-210294 PMC8075566

[34] Santabárbara J, Lipnicki DM, Villagrasa B, et al. Anxiety and risk of dementia: systematic review and meta-analysis of prospective cohort studies. Maturitas. 2019;119:14–20. 10.1016/j.maturitas.2018.10.014. 30502746 10.1016/j.maturitas.2018.10.014

[35] Sun L, Li W, Qiu Q, et al. Anxiety adds the risk of cognitive progression and is associated with axon/synapse degeneration among cognitively unimpaired older adults. EBioMedicine. 2023;94:104703. 10.1016/j.ebiom.2023.104703. 37429081 10.1016/j.ebiom.2023.104703 PMC10435838

[36] Cohen S, van Dyck CH, Gee M, et al. Lecanemab Clarity AD: quality-of-life results from a randomized, double-blind phase 3 trial in early Alzheimer’s disease. J Prev Alzheimers Dis. 2023;10:771–7. 10.14283/jpad.2023.123. 37874099 10.14283/jpad.2023.123

[37] Tysinger B, Wei Y, Heun-Johnson H, et al. Long-term value of lecanemab to individuals and families. Alzheimers Dement. 2025;11:e70151. 10.1002/trc2.70151. 10.1002/trc2.70151 PMC12394178 40895811

[38] Greenberg SM, Bax F, van Veluw SJ. Amyloid-related imaging abnormalities: Manifestations, metrics and mechanisms. Nat Rev Neurol. 2025;21:193–203. 10.1038/s41582-024-01053-8. 39794509 10.1038/s41582-024-01053-8

[39] Hampel H, Elhage A, Cho M, et al. Amyloid-related imaging abnormalities (ARIA): radiological, biological and clinical characteristics. Brain. 2023;146:4414–24. 10.1093/brain/awad188. 37280110 10.1093/brain/awad188 PMC10629981

[40] APA Practice Organization. Psychologists promote health and well-being throughout our nation. Am Psychol Assoc 2011. https://www.apa.org/health/promote-well-being (accessed June 16, 2026)

[41] Wahass SH. The role of psychologists in health care delivery. J Fam Community Med. 2005;12:63–70. PMC3410123 23012077

[42] Pahlke S, Kahale LA, Mahinrad S, et al. Blood-based biomarkers for detecting Alzheimer’s disease pathology in cognitively impaired individuals within specialized care settings: a systematic review and meta-analysis. Alzheimers Dement. 2025;21:e70828. 10.1002/alz.70828. 41193403 10.1002/alz.70828 PMC12590577

[43] Hunter TR, Santos LE, Tovar-Moll F, et al. Alzheimer’s disease biomarkers and their current use in clinical research and practice. Mol Psychiatry. 2025;30:272–84. 10.1038/s41380-024-02709-z. 39232196 10.1038/s41380-024-02709-z

[44] Assfaw AD, Schindler SE, Morris JC. Advances in blood biomarkers for Alzheimer disease (AD): a review. Kaohsiung J Med Sci. 2024;40:692–8. 10.1002/kjm2.12870. 38888066 10.1002/kjm2.12870 PMC11895589

[45] Mielke MM, Fowler NR. Alzheimer disease blood biomarkers: considerations for population-level use. Nat Rev Neurol. 2024;20:495–504. 10.1038/s41582-024-00989-1. 38862788 10.1038/s41582-024-00989-1 PMC11347965

[46] Delaby C, Hirtz C, Lehmann S. Overview of the blood biomarkers in Alzheimer’s disease: promises and challenges. Rev Neurol (Paris). 2023;179:161–72. 10.1016/j.neurol.2022.09.003. 36371265 10.1016/j.neurol.2022.09.003

[47] Hansson O, Blennow K, Zetterberg H, et al. Blood biomarkers for Alzheimer’s disease in clinical practice and trials. Nat Aging. 2023;3:506–19. 10.1038/s43587-023-00403-3. 37202517 10.1038/s43587-023-00403-3 PMC10979350

[48] Atri A, Dickerson BC, Clevenger C, et al. Alzheimer’s Association clinical practice guideline for the diagnostic evaluation, testing, counseling, and disclosure of suspected Alzheimer’s disease and related disorders (DETeCD-ADRD): executive summary of recommendations for primary care. Alzheimers Dement. 2024;21:e14333. 10.1002/alz.14333. 39713942 10.1002/alz.14333 PMC12173843

[49] Bolton CJ, Rostamzadeh A, Chin N, et al. Disclosure of Alzheimer’s disease blood-based biomarker results in a primary care setting: opportunities and challenges. J Prev Alzheimers Dis. 2025;12:100310. 10.1016/j.tjpad.2025.100310. 40713244 10.1016/j.tjpad.2025.100310 PMC12501330

[50] Stites SD, Vogt NM, Blacker D, et al. Patients asking about APOE gene test results? Here’s what to tell them. J Fam Pract. 2022;71:E1-7. 10.12788/jfp.0397. 10.12788/jfp.0397 PMC10032667 35730709

[51] Alzheimer’s Association. 2022 Alzheimer’s disease facts and figures, Special report: More than normal aging: Understanding mild cognitive impairment. Alzheimers Dement J Alzheimers Assoc. 2022;18:700–89. 10.1002/alz.12638.

[52] Alzheimer’s Association. 2025 Alzheimer’s disease facts and figures. Alzheimers Dement. 2025;21:e70235. 10.1002/alz.70235.

[53] Alzheimer’s Association. 2026 Alzheimer’s disease facts and figures. Alzheimers Dement. 2026;22:e71345. 10.1002/alz.71345.

[54] Kinzer A, Suhr JA. Dementia worry and its relationship to dementia exposure, psychological factors, and subjective memory concerns. Appl Neuropsychol Adult. 2016;23:196–204. 10.1080/23279095.2015.1030669. 26496236 10.1080/23279095.2015.1030669

[55] Tang W, Kannaley K, Friedman DB, et al. Concern about developing Alzheimer’s disease or dementia and intention to be screened: An analysis of national survey data. Arch Gerontol Geriatr. 2017;71:43–9. 10.1016/j.archger.2017.02.013. 28279898 10.1016/j.archger.2017.02.013 PMC5995109

[56] Blendon R, Benson J, Wikler E, et al. P4‐395: Five‐country survey of public experiences, attitudes and beliefs concerning Alzheimer’s disease and the value of a diagnosis. Alzheimers Dement 2011;7. 10.1016/j.jalz.2011.09.209.

[57] Roberts JS, McLaughlin SJ, Connell CM. Public beliefs and knowledge about risk and protective factors for Alzheimer’s disease. Alzheimers Dement J Alzheimers Assoc. 2014;10:S381–9. 10.1016/j.jalz.2013.07.001. 10.1016/j.jalz.2013.07.001 PMC4163539 24630852

[58] Werner P. Assessing correlates of concern about developing Alzheimer’s dementia among adults with no family history of the disease. Am J Alzheimers Dis Other Demen. 2002;17:331–7. 10.1177/153331750201700609. 12501479 10.1177/153331750201700609 PMC10833670

[59] Werner P, Goldberg S, Mandel S, et al. Gender differences in lay persons’ beliefs and knowledge about Alzheimer’s disease (AD): a national representative study of Israeli adults. Arch Gerontol Geriatr. 2013;56:400–4. 10.1016/j.archger.2012.11.001. 23219063 10.1016/j.archger.2012.11.001

[60] Alzheimer’s Association. 2020 Alzheimer’s disease facts and figures, Special report: On the front lines: Primary care physicians and Alzheimer’s care in America. Alzheimers Dement J Alzheimers Assoc. 2020;16:63–91. 10.1002/alz.12068.

[61] Hansson O, Edelmayer RM, Boxer AL, et al. The Alzheimer’s Association appropriate use recommendations for blood biomarkers in Alzheimer’s disease. Alzheimers Dement. 2022;18:2669–86. 10.1002/alz.12756. 35908251 10.1002/alz.12756 PMC10087669

[62] Johnson KA, Minoshima S, Bohnen NI, et al. Appropriate use criteria for amyloid PET. Alzheimers Dement J Alzheimers Assoc. 2013;9:e-1-16. 10.1016/j.jalz.2013.01.002. 10.1016/j.jalz.2013.01.002 PMC3733252 23360977

[63] Shaw LM, Arias J, Blennow K, et al. Appropriate use criteria for lumbar puncture and cerebrospinal fluid testing in the diagnosis of Alzheimer’s disease. Alzheimers Dement. 2018;14:1505–21. 10.1016/j.jalz.2018.07.220. 30316776 10.1016/j.jalz.2018.07.220 PMC10013957

[64] Anand S, Schoo C. Mild cognitive impairment. StatPearls, Treasure Island (FL): StatPearls Publishing; 2026. 38261679

[65] Ketchum FB, Chin NA, Grill JD. Aligning Alzheimer disease biology with care. JAMA Neurol. 2025;82:537–8. 10.1001/jamaneurol.2024.5154. 39928326 10.1001/jamaneurol.2024.5154 PMC13402869

[66] Donohue MC, Sperling RA, Petersen R, et al. Association between elevated brain amyloid and subsequent cognitive decline among cognitively normal persons. JAMA. 2017;317:2305–16. 10.1001/jama.2017.6669. 28609533 10.1001/jama.2017.6669 PMC5736301

[67] Roberts RO, Aakre JA, Kremers WK, et al. Prevalence and outcomes of amyloid positivity among persons without dementia in a longitudinal, population-based setting. JAMA Neurol. 2018;75:970–9. 10.1001/jamaneurol.2018.0629. 29710225 10.1001/jamaneurol.2018.0629 PMC6142936

[68] Huszár Z, Engh MA, Pavlekovics M, et al. Risk of conversion to mild cognitive impairment or dementia among subjects with amyloid and tau pathology: A systematic review and meta-analysis. Alzheimers Res Ther. 2024;16:81. 10.1186/s13195-024-01455-2. 38610055 10.1186/s13195-024-01455-2 PMC11015617

[69] Jack CR, Therneau TM, Lundt ES, et al. Long-term associations between amyloid positron emission tomography, sex, apolipoprotein E and incident dementia and mortality among individuals without dementia: hazard ratios and absolute risk. Brain Commun. 2022;4:fcac017. 10.1093/braincomms/fcac017. 35310829 10.1093/braincomms/fcac017 PMC8924651

[70] Deters KD, Napolioni V, Sperling RA, et al. Amyloid PET imaging in self-identified non-hispanic black participants of the Anti-Amyloid in Asymptomatic Alzheimer’s disease (A4) study. Neurology. 2021;96:e1491–500. 10.1212/WNL.0000000000011599. 33568538 10.1212/WNL.0000000000011599 PMC8032379

[71] Daniilidou M, Öhlund-Wistbacka U, Hagman G, et al. Enhancing diagnostic precision in Alzheimer’s disease: impact of comorbidities on blood biomarkers for clinical integration. Alzheimers Dement J Alzheimers Assoc. 2025;21:e70931. 10.1002/alz.70931. 10.1002/alz.70931 PMC12668905 41326959

[72] Stites SD, Lee BN, Rubright JD, et al. Cognitive complaint types can correlate with cognitive testing, perceived stress, and symptom distress in older adults with normal cognition and dementia. Alzheimer Dis Assoc Disord. 2024;38:34–41. 10.1097/WAD.0000000000000595. 38133963 10.1097/WAD.0000000000000595 PMC10922433

[73] Cummings JL, Zhou Y, Yang Y, et al. Alzheimer’s disease drug development pipeline: 2026. Alzheimers Dement Transl Res Clin Interv. 2026;12:e70251. 10.1002/trc2.70251. 10.1002/trc2.70251 PMC13140253 42095064

[74] Murman DL. The impact of age on cognition. Semin Hear. 2015;36:111–21. 10.1055/s-0035-1555115. 27516712 10.1055/s-0035-1555115 PMC4906299

[75] Cognitive Symptoms - NCI 2021. https://www.cancer.gov/rare-brain-spine-tumor/living/symptoms/cognitive (accessed March 24, 2026).

[76] Stites SD, Harkins K, Rubright JD, et al. Relationships between cognitive complaints and quality of life in older adults with mild cognitive impairment, mild Alzheimer disease dementia, and normal cognition. Alzheimer Dis Assoc Disord. 2018. 10.1097/WAD.0000000000000262. 10.1097/WAD.0000000000000262 PMC6249095 29944474

[77] Penninx BWJH, Pine DS, Holmes EA, et al. Anxiety disorders. Lancet. 2021;397:914–27. 10.1016/S0140-6736(21)00359-7. 33581801 10.1016/S0140-6736(21)00359-7 PMC9248771

[78] Langbaum JB, Bradbury AR, Egleston BL, et al. Impact of learning APOE genotype on cognitively unimpaired adults: a pre-screening cohort study of the Alzheimer’s Prevention Initiative Generation Study 1. Lancet Healthy Longev. 2025;6:100778. 10.1016/j.lanhl.2025.100778. 41319675 10.1016/j.lanhl.2025.100778 PMC12703885

[79] Grill JD, Sultzer DL, Burns JM, et al. Impact of disclosing amyloid imaging results to cognitively normal research participants: the A4 experience. Alzheimers Dement J Alzheimers Assoc. 2018;14:P215. 10.1016/j.jalz.2018.06.2336.

[80] Grill JD, Raman R, Ernstrom K, et al. Short-term psychological outcomes of disclosing amyloid imaging results to research participants who do not have cognitive impairment. JAMA Neurol. 2020;77:1504–13. 10.1001/jamaneurol.2020.2734. 32777010 10.1001/jamaneurol.2020.2734 PMC7418046

[81] Stites SD, Dedhia M, Harkins K, et al. Learning about a heightened genetic risk for dementia: expected stigma is greater than experienced stigma. Psychol Aging. 2026. 10.1037/pag0000970.10.1037/pag000097041729767

[82] Stites SD, Dedhia M, Kuz C. Predictors of extreme future time perspective change in persons who learn an Alzheimer’s disease biomarker test result. Aging Ment Health 2026;1–17. 10.1080/13607863.2026.2643904. 10.1080/13607863.2026.2643904 PMC13571108 41870451

[83] Yin S, Xiao J, Zhu X, et al. Improved mood boosts memory training gains in older adults with subjective memory complaints via enhanced amygdala-hippocampal connectivity. Am J Geriatr Psychiatry. 2023;31:808–19. 10.1016/j.jagp.2023.04.003. 37164780 10.1016/j.jagp.2023.04.003

[84] Tonga JB, Eilertsen D-E, Solem IKL, et al. Effect of self-efficacy on quality of life in people with mild cognitive impairment and mild dementia: the mediating roles of depression and anxiety. Am J Alzheimers Dis Dementias®. 2020;35:1533317519885264. 10.1177/1533317519885264. 10.1177/1533317519885264 PMC10623983 31916847

[85] Seignourel PJ, Kunik ME, Snow L, et al. Anxiety in dementia. Clin Psychol Rev. 2008;28:1071–82. 10.1016/j.cpr.2008.02.008. 18555569 10.1016/j.cpr.2008.02.008 PMC2575801

[86] Anantapong K, Jiraphan A, Aunjitsakul W, et al. Behavioural and psychological symptoms of people with dementia in acute hospital settings: a systematic review and meta-analysis. Age Ageing. 2025;54:afaf013. 10.1093/ageing/afaf013. 39888603 10.1093/ageing/afaf013 PMC11784590

[87] Cerejeira J, Lagarto L, Mukaetova-Ladinska EB. Behavioral and psychological symptoms of dementia. Front Neurol. 2012;3:73. 10.3389/fneur.2012.00073. 22586419 10.3389/fneur.2012.00073 PMC3345875

[88] Devshi R, Shaw S, Elliott-King J, et al. Prevalence of behavioural and psychological symptoms of dementia in individuals with learning disabilities. Diagnostics (Basel). 2015;5:564–76. 10.3390/diagnostics5040564. 26854171 10.3390/diagnostics5040564 PMC4728475

[89] Stewart R, Hotopf M, Dewey M, et al. Current prevalence of dementia, depression and behavioural problems in the older adult care home sector: the South East London Care Home Survey. Age Ageing. 2014;43:562–7. 10.1093/ageing/afu062. 24855111 10.1093/ageing/afu062

[90] Aarsland D, Sharp S, Ballard C. Psychiatric and behavioral symptoms in Alzheimer’s disease and other dementias: etiology and management. Curr Neurol Neurosci Rep. 2005;5:345–54. 10.1007/s11910-005-0058-4. 16131417 10.1007/s11910-005-0058-4

[91] Kwak YT, Yang Y, Koo M-S. Anxiety in dementia. Dement Neurocogn Disord. 2017;16:33. 10.12779/dnd.2017.16.2.33. 30906368 10.12779/dnd.2017.16.2.33 PMC6427954

[92] Remes O, Mendes JF, Templeton P. Biological, psychological, and social determinants of depression: A review of recent literature. Brain Sci. 2021;11:1633. 10.3390/brainsci11121633. 34942936 10.3390/brainsci11121633 PMC8699555

[93] Riley RJ, Burgener S, Buckwalter KC. Anxiety and stigma in dementia: a threat to aging in place. Nurs Clin North Am. 2014;49:213–31. 10.1016/j.cnur.2014.02.008. 24846469 10.1016/j.cnur.2014.02.008 PMC4032087

[94] Stites SD, Schumann R, Shi Y, et al. Depression and anxiety modify cognitive concerns associated with amyloid and early clinical impairment. Neuropsychology 2026;Advance online publication. 10.1037/neu0001086. 10.1037/neu0001086 PMC13290126 42295275

[95] Toyoshima K, Ichiki M, Inoue T, et al. The role of cognitive complaints in the relationship between trait anxiety, depressive symptoms, and subjective well-being and ill-being in adult community volunteers. Neuropsychiatr Dis Treat. 2021;17:1299–309. 10.2147/NDT.S303751. 33958871 10.2147/NDT.S303751 PMC8096453

[96] Arborelius L, Owens MJ, Plotsky PM, et al. The role of corticotropin-releasing factor in depression and anxiety disorders. J Endocrinol. 1999;160:1–12. 10.1677/joe.0.1600001. 9854171 10.1677/joe.0.1600001

[97] Jiang X, Xu K, Hoberman J, et al. BDNF variation and mood disorders: a novel functional promoter polymorphism and Val66Met are associated with anxiety but have opposing effects. Neuropsychopharmacology. 2005;30:1353–61. 10.1038/sj.npp.1300703. 15770238 10.1038/sj.npp.1300703

[98] Ressler KJ, Nemeroff CB. Role of serotonergic and noradrenergic systems in the pathophysiology of depression and anxiety disorders. Depress Anxiety. 2000;12(Suppl 1):2–19. 10.1002/1520-6394(2000)12:1+<;2::AID-DA2>;3.0.CO;2-4. 11098410 10.1002/1520-6394(2000)12:1+<2::AID-DA2>3.0.CO;2-4

[99] Hao L, Wang X, Zhang L, et al. Prevalence, risk factors, and complaints screening tool exploration of subjective cognitive decline in a large cohort of the Chinese population. J Alzheimers Dis JAD. 2017;60:371–88. 10.3233/JAD-170347. 28869471 10.3233/JAD-170347

[100] Poppe M, Duffy L, Marchant NL, et al. The APPLE Tree programme: Active Prevention in People at risk of dementia through Lifestyle, bEhaviour change and Technology to build REsiliEnce—randomised controlled trial. Trials. 2022;23:596. 10.1186/s13063-022-06557-6. 35883143 10.1186/s13063-022-06557-6 PMC9315085

[101] Hummel J, Weisbrod C, Boesch L, et al. AIDE–acute illness and depression in elderly patients. Cognitive behavioral group psychotherapy in geriatric patients with comorbid depression: a randomized, controlled trial. J Am Med Dir Assoc. 2017;18:341–9. 10.1016/j.jamda.2016.10.009. 27956074 10.1016/j.jamda.2016.10.009

[102] Martino-Adami PV, Jessen F, Brosseron F, et al. Exploring blood-based biomarkers in late-life depression: correlates of psychotherapeutic treatment outcomes. Eur Psychiatry. 2026;69:e18. 10.1192/j.eurpsy.2026.10153. 41572662 10.1192/j.eurpsy.2026.10153 PMC12925672

[103] Gomoll BP, Kumar A. Managing anxiety associated with neurodegenerative disorders. F1000Prime Rep 2015;7. 10.12703/P7-05. 10.12703/P7-05 PMC4311274 25705388

[104] Kwok JYY, Kwan JCY, Auyeung M, et al. Effects of mindfulness yoga vs stretching and resistance training exercises on anxiety and depression for people with Parkinson disease: a randomized clinical trial. JAMA Neurol. 2019;76:755–63. 10.1001/jamaneurol.2019.0534. 30958514 10.1001/jamaneurol.2019.0534 PMC6583059

[105] Zhang W, Xiao D, Mao Q, et al. Role of neuroinflammation in neurodegeneration development. Signal Transduct Target Ther. 2023;8:267. 10.1038/s41392-023-01486-5. 37433768 10.1038/s41392-023-01486-5 PMC10336149

[106] Eggers S, Hoggarth ZE, Nagdeo K, et al. Food insecurity modifies the association between the gut microbiome and the risk of cognitive impairment in adults. Npj Aging. 2025;11:47. 10.1038/s41514-025-00241-0. 40533471 10.1038/s41514-025-00241-0 PMC12177064

[107] McMichael AJ, McGuinness B, Lee J, et al. Food insecurity and brain health in adults: A systematic review. Crit Rev Food Sci Nutr. 2022;62:8728–43. 10.1080/10408398.2021.1932721. 34047662 10.1080/10408398.2021.1932721

[108] Medoro A, Scapagnini G, Hu FB, et al. The relationship between dietary patterns and neuroinflammation. Crit Rev Food Sci Nutr. 2026;0:1–18. 10.1080/10408398.2026.2645262. 10.1080/10408398.2026.2645262 41854289

[109] Smith L, López Sánchez GF, Shin JI, et al. Food insecurity and subjective cognitive complaints among adults aged ≥ 65 years from low- and middle-income countries. Eur J Nutr. 2023;62:3217–26. 10.1007/s00394-023-03226-5. 37550594 10.1007/s00394-023-03226-5 PMC10611875

[110] Stites SD, Milne R, Karlawish J. Advances in Alzheimer’s imaging are changing the experience of Alzheimer’s disease. Alzheimers Dement. 2018;10:285–300. 10.1016/j.dadm.2018.02.006. 10.1016/j.dadm.2018.02.006 PMC5956938 29780873

[111] Tao Q-Q, Lin R-R, Wu Z-Y. Early diagnosis of Alzheimer’s disease: Moving toward a blood-based biomarkers era. Clin Interv Aging. 2023;18:353–8. 10.2147/CIA.S394821. 36911809 10.2147/CIA.S394821 PMC10001034

[112] Hartz SM, Goswami S, Oliver A, et al. What is the psychological and cognitive impact of returning Alzheimer disease dementia research results to healthy research participants? A delayed-start randomised clinical trial protocol for the WeSHARE study (Washington University study of having Alzheimer disease research results explained). BMJ Open. 2026;16:e099970. 10.1136/bmjopen-2025-099970. 10.1136/bmjopen-2025-099970 PMC12778337 41500647

[113] Largent EA, Grill JD, O’Brien K, et al. Testing for Alzheimer disease biomarkers and disclosing results across the disease continuum. Neurology. 2023;100:1010–9. 10.1212/WNL.0000000000206891. 36720642 10.1212/WNL.0000000000206891 PMC10238153

[114] Milne R, Bunnik E, Tromp K, et al. Ethical issues in the development of readiness Cohorts in Alzheimer’s disease research. J Prev Alzheimers Dis. 2017;4:125–31. 10.14283/jpad.2017.5. 29186282 10.14283/jpad.2017.5

[115] Rico V, Zelinsky M, Ford PJ, et al. Recommended approaches to sharing individual research results in Alzheimer’s disease research: a multidisciplinary expert Delphi consensus. J Alzheimers Dis. 2025;108:703–18. 10.1177/13872877251379076. 40997223 10.1177/13872877251379076 PMC13001161

[116] Marteau TM, Bekker H. The development of a six-item short-form of the state scale of the Spielberger state-trait anxiety inventory (STAI). Br J Clin Psychol. 1992;31:301–6. 10.1111/j.2044-8260.1992.tb00997.x. 1393159 10.1111/j.2044-8260.1992.tb00997.x

[117] Beck AT, Epstein N, Brown G, et al. An inventory for measuring clinical anxiety: psychometric properties. J Consult Clin Psychol. 1988;56:893–7. 10.1037/0022-006x.56.6.893. 10.1037//0022-006x.56.6.893 3204199

[118] Zigmond AS, Snaith RP. The hospital anxiety and depression scale. Acta Psychiatr Scand. 1983;67:361–70. 10.1111/j.1600-0447.1983.tb09716.x. 6880820 10.1111/j.1600-0447.1983.tb09716.x

[119] Yesavage JA, Brink TL, Rose TL, et al. Development and validation of a geriatric depression screening scale: a preliminary report. J Psychiatr Res. 1982;17:37–49. 10.1016/0022-3956(82)90033-4. 7183759 10.1016/0022-3956(82)90033-4

[120] Kroenke K, Spitzer RL, Williams JBW. The PHQ-9: validity of a brief depression severity measure. J Gen Intern Med. 2001;16:606–13. 10.1046/j.1525-1497.2001.016009606.x. 11556941 10.1046/j.1525-1497.2001.016009606.x PMC1495268

[121] Beck AT, Ward CH, Mendelson M, et al. An inventory for measuring depression. Arch Gen Psychiatry. 1961;4:561–71. 10.1001/archpsyc.1961.01710120031004. 13688369 10.1001/archpsyc.1961.01710120031004

[122] Posner K, Brown GK, Stanley B, et al. The Columbia-Suicide Severity Rating Scale: initial validity and internal consistency findings from three multisite studies with adolescents and adults. Am J Psychiatry. 2011;168:1266–77. 10.1176/appi.ajp.2011.10111704. 22193671 10.1176/appi.ajp.2011.10111704 PMC3893686

[123] Cummings J. The Neuropsychiatric Inventory: development and applications. J Geriatr Psychiatry Neurol. 2020;33:73–84. 10.1177/0891988719882102. 32013737 10.1177/0891988719882102 PMC8505128

[124] Brodaty H, Chau T, Heffernan M, et al. An online multidomain lifestyle intervention to prevent cognitive decline in at-risk older adults: a randomized controlled trial. Nat Med. 2025;31:565–73. 10.1038/s41591-024-03351-6. 39875685 10.1038/s41591-024-03351-6

[125] Burke AD, Goldfarb D, Bollam P, et al. Diagnosing and treating depression in patients with Alzheimer’s disease. Neurol Ther. 2019;8:325–50. 10.1007/s40120-019-00148-5. 31435870 10.1007/s40120-019-00148-5 PMC6858899

[126] Byers AL, Yaffe K. Depression and risk of developing dementia. Nat Rev Neurol. 2011;7:323–31. 10.1038/nrneurol.2011.60. 21537355 10.1038/nrneurol.2011.60 PMC3327554

[127] García-Alberca JM. Cognitive-behavioral treatment for depressed patients with Alzheimer’s disease. An Open Trial Arch Gerontol Geriatr. 2017;71:1–8. 10.1016/j.archger.2017.02.008. 28237746 10.1016/j.archger.2017.02.008

[128] Modrego PJ, Cerio LDD, Lobo A. The interface between depression and Alzheimer’s disease. A comprehensive approach. Ann Indian Acad Neurol. 2023;26:315–25. 10.4103/aian.aian_326_23. 37970263 10.4103/aian.aian_326_23 PMC10645209

[129] Tetsuka S. Depression and dementia in older adults: a neuropsychological review. Aging Dis. 2021;12:1920. 10.14336/AD.2021.0526. 34881077 10.14336/AD.2021.0526 PMC8612610

[130] Leyhe T, Reynolds CF, Melcher T, et al. A common challenge in older adults: classification, overlap, and therapy of depression and dementia. Alzheimers Dement. 2017;13:59–71. 10.1016/j.jalz.2016.08.007. 27693188 10.1016/j.jalz.2016.08.007

[131] American Psychological Association. Geriatric Depression Scale (GDS). Am Psychol Assoc 2020. https://www.apa.org/pi/about/publications/caregivers/practice-settings/assessment/tools/geriatric-depression#%3A~%3Atext%3DConstruct%3A%20Depressive%20symptoms%2C.%2C%202017%2C%20respectively%29.

[132] Uomoto KE. Increasing identification and follow-up of older adult depression in primary care. J Prim Care Community Health. 2023;14:21501319231152760. 10.1177/21501319231152758. 36760105 10.1177/21501319231152758 PMC9926000

[133] Weintraub D, Oehlberg KA, Katz IR, et al. Test characteristics of the 15-item geriatric depression scale and Hamilton depression rating scale in Parkinson Disease. Am J Geriatr Psychiatry Off J Am Assoc Geriatr Psychiatry. 2006;14:169–75. 10.1097/01.JGP.0000192488.66049.4b. 10.1097/01.JGP.0000192488.66049.4b PMC1571046 16473982

[134] Liew TM. Symptom clusters of neuropsychiatric symptoms in mild cognitive impairment and their comparative risks of dementia: a cohort study of 8530 older persons. J Am Med Dir Assoc. 2019;20:1054.e1-1054.e9. 10.1016/j.jamda.2019.02.012. 10.1016/j.jamda.2019.02.012 PMC6663577 30926409

[135] Julian LJ. Measures of anxiety: State‐Trait Anxiety Inventory (STAI), Beck Anxiety Inventory (BAI), and Hospital Anxiety and Depression Scale‐Anxiety (HADS‐A). Arthritis Care Res 2011;63. 10.1002/acr.20561. 10.1002/acr.20561 PMC3879951 22588767

[136] Avinir A, Dar S, Taler M, et al. Keeping it simple: mental health assessment in the Gastroenterology Department – using the Hospital Anxiety and Depression Scale (HADS) for IBD patients in Israel. Ther Adv Gastroenterol. 2022;15:17562848211066440. 10.1177/17562848211066439. 35251306 10.1177/17562848211066439 PMC8891839

[137] Michopoulos I, Douzenis A, Kalkavoura C, et al. Hospital Anxiety and Depression Scale (HADS): validation in a Greek general hospital sample. Ann Gen Psychiatry. 2008;7:4. 10.1186/1744-859X-7-4. 18325093 10.1186/1744-859X-7-4 PMC2276214

[138] Turk DC, Dworkin RH, Trudeau JJ, et al. Validation of the Hospital Anxiety and Depression Scale in patients with acute low back pain. J Pain. 2015;16:1012–21. 10.1016/j.jpain.2015.07.001. 26208762 10.1016/j.jpain.2015.07.001

[139] Vodermaier A, Millman RD. Accuracy of the hospital anxiety and depression scale as a screening tool in cancer patients: a systematic review and meta-analysis. Database Abstr. Rev. Eff. DARE Qual.-Assess. Rev. Internet, Centre for Reviews and Dissemination (UK); 2011. 10.1007/s00520-011-1251-4 21898134

[140] Sun Y, Kong Z, Song Y, et al. The validity and reliability of the PHQ-9 on screening of depression in neurology: a cross sectional study. BMC Psychiatry. 2022;22:98. 10.1186/s12888-021-03661-w. 35139810 10.1186/s12888-021-03661-w PMC8827244

[141] University of Washington, Department of Psychiatry and Behavioral Sciences, AIMS Center. Phq-9 Depression Scale Questionnaire. AIMS Cent 2024. https://aims.uw.edu/resource/phq-9-depression-scale/.

